# Prognostic Factors of Hodgkin's Disease Recurrence: An Experience From a Tertiary Academic Center in Iran

**DOI:** 10.7759/cureus.75710

**Published:** 2024-12-14

**Authors:** Sharareh Seifi, Zahra Esfahanimonfared, Adnan Khosravi, Fatemeh Moeini Nia

**Affiliations:** 1 Research Center of Thoracic Oncology (RCTO), National Research Institute of Tuberculosis and Lung Disease (NRITLD), Tehran, IRN; 2 Medicine, NYC Health + Hospitals/South Brooklyn Health, New York, USA

**Keywords:** clinical characteristics, hodgkin's disease (hd), prognostic risk factors, progression-free survival, recurrence predictors

## Abstract

Introduction

Despite the favorable prognosis of Hodgkin's disease (HD), some patients experience disease recurrence. Therefore, determining recurrence prognostic factors is very crucial to identify patients at risk of early relapse, maintain remission, and improve outcomes.

Materials and methods

This retrospective cohort study enrolled HD patients at the National Research Institute of Tuberculosis and Lung Disease (NRITLD), Masih Daneshvari Hospital, between January 1, 2002, and June 30, 2018. Demographic characteristics and clinical outcomes, including response to treatment, disease progression, and survival data, were considered.

Results

Two hundred fifty patients were enrolled. The mean age at diagnosis was 30.03 years. The female-to-male ratio was 1.06:1. Eighty-five (34%) disease recurrences occurred within the study period, most occurring before three years of initial diagnosis. The mean of progression-free survival (PFS) was 18.4 months. In both univariate and multivariate analyses, the advanced stage (stage IV) was significantly associated with shorter PFS (P=0.0001 and P=0.004, respectively). Also, shorter PFS was observed in the presence of bulky tumors (P=0.037/univariate analysis) and high-risk International Prognostic Score (IPS) (P=0.030/multivariate analysis).

Conclusion

Our findings in the Iranian HD patient cohort revealed that the risk of disease recurrence is higher in the presence of some clinical characteristics such as bulky disease, higher IPS, and advanced stage which needs special attention. Close follow-up, especially within three years after initial diagnosis, has a great importance to detect early relapse.

## Introduction

Hodgkin's disease (HD) is a B-cell-derived malignancy with epidemiologic heterogeneity [1]. The exact mechanisms of HD pathogenesis are relatively unknown and thought to be associated with genetic susceptibility and environmental risk factors [2]. The presence of large Reed-Sternberg (RS) cells and abundant reactive cells in the tumor microenvironment are the main histopathologic characteristics of this disease [3]. Recently, imperfect B-cell expression program is considered as a probable pathophysiology of some subtypes of HD [4]. Now, by combined modality therapy (chemotherapy and/or radiotherapy), HD is known as a disease with a favorable prognosis and high cure rate [5]. Unfortunately, some patients still experience disease recurrence, especially during three years of disease diagnosis. Salvage second-line chemotherapy or radiotherapy produces a low remission rate in disease recurrence. Therefore, consideration of relapse prognostic factors is crucial for the early identification of patients at high risk of recurrence and appropriate treatment plans. This study aimed to describe HD patients' demographic outcomes and probable relapse prognostic factors at a tertiary care hospital in Iran.

## Materials and methods

This retrospective cohort study with 250 HD patients was conducted at the National Research Institute of Tuberculosis and Lung Disease (NRITLD), Masih Daneshvari Hospital, in Tehran, Iran, between January 1, 2002, and June 30, 2018. Disease histologies were confirmed according to the classification guidelines of the World Health Organization (WHO) [6] for all the study patients. Approval for this study was obtained from Shahid Beheshti University of Medical Sciences Ethics and Local Scientific Committees (approval number: IR.SBMU.NRITLD.REC.1403.099), and this study was conducted in compliance with the Helsinki Declaration.

Based on the WHO/Revised European-American Lymphoma (REAL) classification [6,7], HD is divided into two main histological subgroups: classical HD (including nodular sclerosis, mixed cellularity, lymphocyte rich, and lymphocyte depleted) and nodular lymphocyte predominant (NLP). Classical HD "not otherwise specified" was used whenever none of the classical HD subgroups was defined.

The B symptoms are defined in the presence of any fever (>38°C), drenching night sweats, and weight loss (at least 10% of body weight over six months). The bulky disease was defined as a mass larger than 10 cm or greater than a third of the transthoracic diameter at any level of the thoracic vertebrae. The treatment efficacy was assessed after every two cycles of chemotherapy. Response rate (RR) was evaluated according to the "Response Evaluation Criteria in Solid Tumors" (RECIST) criteria [8]. The objective response rate (ORR) is defined as the sum of the number of complete responses (CR) and partial responses (PR). The International Prognostic Score (IPS) based on seven clinical parameters (male sex, age >45 years, stage IV disease, hemoglobin <10.5 g/dl, white blood cell (WBC) count ≥15×109/L, lymphocyte count <0.6×109/L or <8% of total WBC count, and albumin <40 g/L: each one defined as 1 score) according to the Hasenclever and Diehl study was considered [9]. We divided patients according to IPS into a low-risk group (IPS 0, 1, and 2) and a high-risk group (other scores). Refractory HD is defined by a less than 50% decrease in lesion size with treatment in the absence of new lesion development after 90 days of completing treatment [10]. The progressive disease usually manifests as the appearance of any new lesion, a 50% increase in the longest diameter of a previously identified lesion, or new/recurrent involvement of the bone marrow. Relapsed disease reflects the appearance of any new lesion after treatment and initial complete remission. We chose 45-year-olds as the cut-point in our analysis as IPS has been determined [9].

Human immunodeficiency virus (HIV) and hepatitis A, B, and C virological tests were performed before treatment. All patients initially had been treated with an ABVD regimen (Adriamycin 25 mg/m^2^, bleomycin 10 mg/m^2^, vinblastine 6 mg/m^2^, and dacarbazine 375 mg/m^2^ every two weeks up to eight cycles). Also, radiotherapy was done following chemotherapy for bulky tumors or residual disease. Patients who had progressive or refractory disease at any stage received salvage regimens. We evaluated the correlation of relapse with clinical and demographic characteristics.

Positron emission tomography (PET) studies were not available for all patients during the study period in our country; thus, computed tomography (CT) was applied for staging and response evaluation.

For the primary analysis, a minimum of six months of enrollment data before and after the index date was required.

Antibodies and immunoperoxidase staining

The previously stained slides for CD30 of these blocks were reviewed for the unequivocal identification of neoplastic cells. All sections were stained with antibodies against leukocyte common antigen (LCA), CD3, CD20, CD15, and CD30. Hodgkin's and RS cells in reactive lymphocyte background with any degree of nuclear staining were considered positive, and the percentage was calculated.

Statistical analysis

The mean±standard deviation (SD) was calculated for the continuous variable. For categorical values, numbers and percentages were obtained. Progression-free survival (PFS) was defined as the time from diagnosis to documented clinical progression or death for any cause. Patients who were alive or lost to follow-up at the time of data analysis were censored for PFS analysis. Kaplan-Meier's survival curves were obtained for PFS. To compare the frequencies between different groups, the chi-squared test was used. A P-value of less than 0.05 was considered statistically significant. The Cox proportional hazards model was utilized for both univariate and multivariate analyses assessing the impact of independent variables (including age, gender, disease stage, presence or absence of B symptoms, subtypes of groups, and IPS) on PFS. All confidence intervals (CIs) for parameters to be estimated were constructed with a significance level of alpha=0.05. IBM SPSS Statistics for Windows, V. 19.0 (Released 2010, IBM Corp., Armonk, NY, USA), was used for data analysis. Follow-up time was defined from the time of diagnosis until censoring (at death or follow-up loss) or last visit.

## Results

The mean (SD) age at diagnosis was 30.03 (11.1) years (median age: 27 years, range: 13-71). There was a slight female (51.6%) predominance over males (48.4%). The female-to-male ratio was 1.06:1. The mean follow-up time was 51.5 months. Bone marrow biopsy was done in 68.8% (n=172) of patients, and among them, bone marrow involvement was documented in 16 cases (6.4%). Concurrent tuberculosis (TB) and HIV/acquired immunodeficiency syndrome (AIDS) were observed in two (1%) and one (0.5%) patients, respectively. At the time of data analysis, secondary malignancy was not seen in any patients. The distribution of demographic characteristics is summarized in Table 1.

**Table 1:** Demographic and clinical characteristics of study patients. ^a^Stage according to the Cotswolds-modified Ann Arbor staging system for Hodgkin's lymphoma. ^b^Defined as the sum of the number of complete response and partial response. NS: nodular sclerosis; MC: mixed cellularity; NLP: nodular lymphocyte predominant-type Hodgkin's lymphoma; IPS: International Prognostic Score; ORR: objective response rate; SD: stable disease; NA: not assessed

	Number (%)
Age	
<45	224 (89.6)
≥45	26 (10.4)
Sex	
Male	121 (48.4)
Female	129 (51.6)
Stage^a^	
I	19 (7.6)
II	117 (48.7)
III	48 (19.1)
IV	66 (26.3)
B symptom presence	
Yes	193 (77.2)
No	57 (22.8)
Bulky disease	
Yes	87 (34.8)
No	163 (65.2)
Subtype	
NS	145 (58)
MC	32 (12.8)
Lymphocyte rich	3 (1.2)
Syncytial variant	2 (0.8)
Not otherwise	67 (26.8)
NLP	1 (0.4)
IPS	
Low risk (0, 1, 2)	150 (60)
High risk (3-7)	100 (40)
Response to treatment	
ORR^b^	215 (86)
SD	8 (3.2)
Progressive	8 (3.2)
NA	19 (7.6)
Progression	
Yes	85 (34)
No	144 (57.6)
NA	21 (8.4)
Outcome	
Alive	139 (55.6)
Dead	26 (10.4)
NA	85 (34)

Treatment response and efficacy

At the time of primary treatment evaluation, 92 patients (36.8%) achieved CR, 123 (49.2%) PR, and eight (3.2%) stable disease (SD), and disease progression was documented in eight cases (3.2%). Response evaluation was not possible in 23 patients (9.2%). Primary site radiotherapy was performed in 32.4% (n=81).

Disease progression

**Figure 1:**
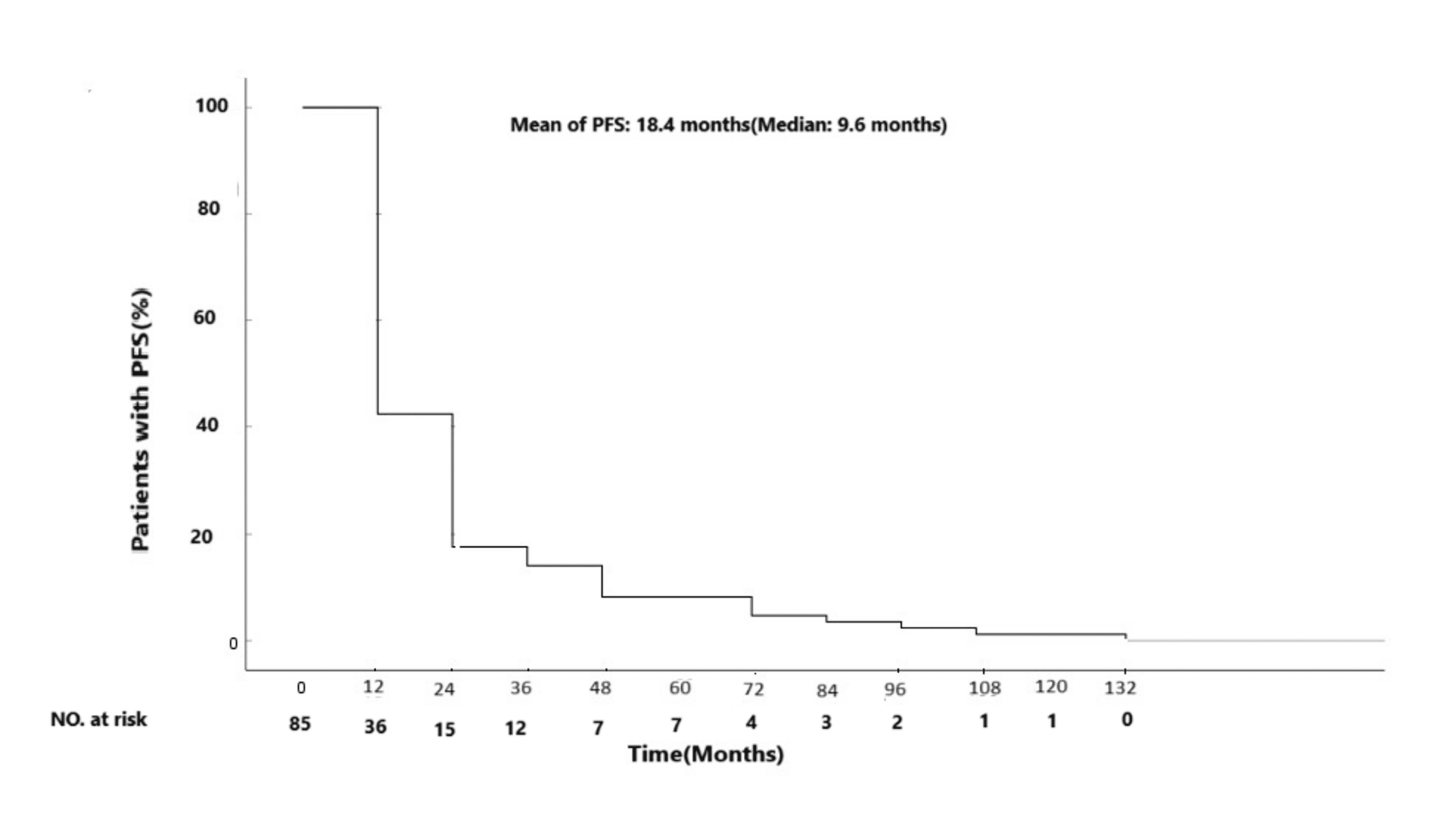
Kaplan-Meier estimates of PFS in patients with progression, recurrence, or death. PFS: progression-free survival

Disease recurrence was documented in 85 (34%) patients. Most of the disease relapse occurred before three years of initial diagnosis in 84.7% (n=72) and the rest of them (n=13, 15.3%) after three years (Figure 1). The mean of PFS was 18.4±2.6 months (median=9.6). The mean of PFS was 17.8, 21.5, 25.3, and 11.9 months in patients with stages I, II, III, and IV, respectively (P=0.186). At the time of disease relapse or in refractory disease, bone marrow transplantation (BMT) was done only in nine (3.6%) patients, and the rest of them were treated with salvage chemotherapy regimens and radiotherapy if applicable. Notably, none of the patients who underwent BMT experienced secondary relapse. Interestingly, among the patients, there were two sisters, and both showed progressive disease during primary treatment evaluation. For one of them, BMT was done, and unfortunately, another one expired during salvage chemotherapy. At the time of data analysis, the death of 27 patients was unfortunately documented (2/165 vs 25/85 deaths in patients without vs with disease progression).

As outlined in Table 2, prognostic factors that are probably related to recurrence were evaluated in both univariate and multivariate analyses by the Cox regression model. Stage IV of the disease was significantly associated with shorter PFS than other stages in both univariate and multivariate analyses (P=0.0001 and P=0.004, respectively). Shorter PFS was seen in patients with bulky tumors (only in multivariate analysis, P=0.037) and high-risk IPS (only in univariate analysis, P=0.030).

**Table 2:** Estimates of PFS regarding prognostic factors. *Significant P-value. ^a^Stage according to the Cotswolds-modified Ann Arbor staging system for Hodgkin's lymphoma. PFS: progression-free survival; SD: standard deviation; CI: confidence interval; NS: nodular sclerosis; IPS: International Prognostic Score

	Estimate of PFS±SD (months)	Univariate analysis (95% CI)	P-value	Multivariate analysis (95% CI)	P-value
Age					
<45 vs ≥45	17.7±2.7 vs 24.3±9.3	0.490-2.83	0.714	0.448-2.917	0.779
Sex					
Male vs female	25.6±4.9 vs12.5±2.3	0.472-1.351	0.402	0.422-1.304	0.3
Stage^a^					
I, II, and III vs IV	23.07±4.07 vs 11.7±2.3	0.201-0.636	0.0001*	0.174-0.724	0.004*
B symptom presence					
Yes vs no	16.1±2.3 vs 28.01±9.07	0.746-2.729	0.284	0.555-2.262	0.75
Bulky disease					
Yes vs no	14.1±2.9 vs 21.3±3.9	0.884-2.617	0.13	1.039-3.542	0.037*
Subtype					
NS vs others	18.3±3.2 vs 21.65±6	0.836-1.105	0.576	0.471-1.456	0.513
IPS					
Low risk (0, 1, 2) vs high risk (3-7)	20.7±3.8 vs 16±3.6	1.059-3.067	0.030*	0.400-1.577	0.511

## Discussion

Our study provides the clinical and pathological characteristics as well as treatment outcomes and relapse prognostic factors of HD in a large cohort of Iranian patients. As we know, limited studies in Iran investigated the association of demographic/clinical factors with HD relapse. To investigate prognostic factors at diagnosis, we noticed that stage IV, bulky disease, and high-risk IPS were associated with a worse prognosis. Thus, these factors need special notice because they can change disease scenarios.

Neither age at diagnosis nor sex had a significant effect on PFS in our series. In our cohort, most patients were female. We believe it needs more investigation to understand the cause of this observation. The male-to-female ratio varies by both age and subtype; therefore, maybe some further subgroup analysis in future studies may be needed. HD most occurs across the bimodal pattern of age: young adults 20-40 and over 55 years old [11]. The majority of our patients were less than 45 years old at diagnosis that is concordant with other studies [12]. The mean age in our study was lower than in some Western countries and the USA but is very similar in other studies in Saudi Arabia [13] and India [14]. Some investigators showed older patients have shorter PFS and survival [15]. It may be due to the increasing age (and/or age-related comorbidities) which augments the adverse events of therapeutic agents. Weaker immune tolerance in the elderly may be another reason. Our result did not support poorer outcomes in patients above 45 years old of a limited number of this group.

Globally, HD is more common in men than women [16]. Other studies in Armenia [17] and Iraq [18] reported a female-predominant pattern in their study. This discrepancy may be correlated to geographical region and/or female-to-male proportion differences in the whole adult population. Most clinicians have a consensus that male patients with HD have a poorer outcome than females [15,19]. They concluded that such differences may be due to different cytotoxic drug metabolism, which can affect treatment response and disease outcome. The male gender has no negative impact on PFS in our cohort.

The nodular sclerosis subtype was the most frequent subtype in our study which is similar to the other geographical regions [18,20]. We did not compare the characteristics and prognosis of classical vs non-classical HD because only one patient in our cohort has an NLP diagnosis.

Differences in terms of treatment efficacy among studies may be explained by different scheduled treatment protocols based on drug availability, physician experience/preference, and, also, patient population characteristics. The complete RR in our study is almost in line with Iraj's study in our country [21]. The CR rate in the mentioned study in patients treated with ABVD regimens was 56.3%. The overall RR in the study of Bhurani et al. [22] was 92.73% which is partially similar to our result. The higher proportion of patients with advanced stages (III and IV), as well as patients with bulky disease, may be probable causes of lower RR in comparison with other studies. Another reason for the lower CR rate and PFS with other studies might be related to the fact that the primary site of involvement of most patients in our study was in the mediastinum. In addition, at the time of the study period, PET scan was not available in Iran; therefore, some of the residual disease after tumor shrinkage (defined as PR) might be non-avid residue/scar. According to the study of Cionini et al. [23], mediastinal involvement was higher in women and in those with NS histopathology, aged younger than 36 years old, and with the presence of B symptoms than in patients with a normal mediastinum. They also concluded that mediastinal adenopathy at disease diagnosis increases the chance of pulmonary involvement. These findings are in line with our result and probably might be an explanation for the higher percentage of women as well as the lower RR and shorter PFS in our study.

Disease relapse following an initial chemotherapy occurs in 10-30% of patients [24] which is almost similar to our result. Most disease recurrence occurs within three years after initial diagnosis [15,25] as our study result showed. The main probable cause of late relapse may be confirmed in this matter in which clinical cure does not always mean the eradication of all the malignant cells. This fact is documented in autopsies of HD long survivors who died of apparently unrelated causes. Sociocultural and financial issues might lead to suboptimal results such as lower PFS and RR. More studies focusing on these factors should be conducted.

Disease extension in HD has two reprehension clinical signs: bulky disease and/or disease stage. The bulky disease may be an indicator of tumor burden. The presence of a bulky tumor is one of the risk factors in the European Organisation for Research and Treatment of Cancer/Groupe d'Etude des Lymphomes de l'Adulte (EORTC/GELA) and the German Hodgkin Study Group (GHSG) stratification scores for HD [26]. Given that there is stronger evidence of advanced-stage PFS than bulky tumors, the advanced stage's role seems to be more important. It may be described by this matter that in the presence of diffuse disease, underestimation of tumor bulk is very likely. Our results strongly support the relation between advanced-stage disease and shorter PFS and poorer outcomes.

Historically, risk stratification by IPS has an important role in determining the escalation of treatment in patients at increased risk for disease relapse. High-risk IPS and advanced-stage disease were associated with poorer PFS in the studies of Biasoli et al. [27] and Shafi et al. [28] as our result demonstrated.

Most studies showed the lengthening of survival in HD recurrence with intensive salvage chemotherapy followed by BMT [29]. Our result showed that neither of the patients who underwent BMT had the other recurrence and all of them were alive at the time of data analysis.

Secondary malignancy was commonly seen in association with HD but neither of our cases showed secondary malignancy.

Familial HD provided a probable scenario in favor of shared prognostic risk factors. It may reflect a genetic role in disease pathogenesis. There were two sisters in the population study who progressed during study time.

Overall, immunosuppressed HD patients are those who are HIV positive and have advanced-stage disease, "B" symptoms, extranodal involvement, and poorer outcomes [30]. Our case of HD/HIV was a 32-year-old man in stage IVB, and in two years of follow-up, no disease recurrence was documented.

Some new therapies such as programmed death ligand 1 (PD-L1) inhibitors [24] were tested following relapse. Our experience with these drugs is very limited because they are not affordable in the vast of our patient population. Currently, advances in antibody therapy made great improvements in the era of HD outcomes introduced. Other ongoing promising treatments included vaccine therapies, checkpoint inhibitors, and cytotoxic T lymphocyte implications. In addition, cell-free tumor DNA may be routinely implicated as a useful tool for HD surveillance in real clinical practice.

Our study had some limitations. First, the retrospective nature of the study may cause significant bias. In addition, the lack of PET scan staging is another limitation of our study. Also, other important risk factors such as environmental factors, occupational exposure, socioeconomic status, lifestyle factors, genetic predisposing factors, and Epstein-Barr virus (EBV) presence were not considered.

## Conclusions

Our findings in the Iranian HD patient cohort revealed that the risk of disease recurrence is higher in the presence of some clinical characteristics such as bulky disease, higher IPS, and advanced stage. Individualized treatment according to risk profile helps to optimize treatment strategies to achieve more favorable outcomes. Close follow-up, especially three years after initial diagnosis, is very necessary to detect early relapse. By ongoing studies, testing novel agents, and new prognostic markers, improvement in relapsed and refractory HD outcomes may be achieved.

## References

[1] Saarinen S, Pukkala E, Vahteristo P, Mäkinen MJ, Franssila K, Aaltonen LA (2013). High familial risk in nodular lymphocyte-predominant Hodgkin lymphoma. J Clin Oncol.

[2] Nakatsuka S, Aozasa K (2006). Epidemiology and pathologic features of Hodgkin lymphoma. Int J Hematol.

[3] Yang M, Ping L, Liu W (2019). Clinical characteristics and prognostic factors of primary extranodal classical Hodgkin lymphoma: a retrospective study. Hematology.

[4] Mata E, Díaz-López A, Martín-Moreno AM (2017). Analysis of the mutational landscape of classic Hodgkin lymphoma identifies disease heterogeneity and potential therapeutic targets. Oncotarget.

[5] Lees C, Keane C, Gandhi MK, Gunawardana J (2019). Biology and therapy of primary mediastinal B-cell lymphoma: current status and future directions. Br J Haematol.

[6] Swerdlow SH, Campo E, Pileri SA (2016). The 2016 revision of the World Health Organization classification of lymphoid neoplasms. Blood.

[7] Lister TA, Crowther D, Sutcliffe SB (1989). Report of a committee convened to discuss the evaluation and staging of patients with Hodgkin's disease: Cotswolds meeting. J Clin Oncol.

[8] Eisenhauer EA, Therasse P, Bogaerts J (2009). New response evaluation criteria in solid tumours: revised RECIST guideline (version 1.1). Eur J Cancer.

[9] Hasenclever D, Diehl V (1998). A prognostic score for advanced Hodgkin's disease. International prognostic factors project on advanced Hodgkin's disease. N Engl J Med.

[10] Cuccaro A, Bartolomei F, Cupelli E, Galli E, Giachelia M, Hohaus S (2014). Prognostic factors in hodgkin lymphoma. Mediterr J Hematol Infect Dis.

[11] Shanbhag S, Ambinder RF (2018). Hodgkin lymphoma: a review and update on recent progress. CA Cancer J Clin.

[12] Major A, Jackson MW, Smith DE, Kamdar M, Rabinovitch R (2019). Inferior outcomes and treatment disparities in elderly patients with classical Hodgkin lymphoma: a national cancer data base analysis. Leuk Lymphoma.

[13] Al-Diab AI, Siddiqui N, Sogiawalla FF, Fawzy EM (2003). The changing trends of adult Hodgkin's disease in Saudi Arabia. Saudi Med J.

[14] Konkay K, Paul TR, Uppin SG, Rao DR (2016). Hodgkin lymphoma: a clinicopathological and immunophenotypic study. Indian J Med Paediatr Oncol.

[15] Huang J, Pang WS, Lok V (2022). Incidence, mortality, risk factors, and trends for Hodgkin lymphoma: a global data analysis. J Hematol Oncol.

[16] Aslani A, Morsali S, Mousavi SE, Choupani S, Yekta Z, Nejadghaderi SA (2024). Adult Hodgkin lymphoma incidence trends in the United States from 2000 to 2020. Sci Rep.

[17] Avagyan A, Danielyan S, Voskanyan A (2016). Treating adults with Hodgkin lymphoma in the developing world: a hospital-based cohort study from Armenia. Asian Pac J Cancer Prev.

[18] Shamoon RP, Ali MD, Shabila NP (2018). Overview and outcome of Hodgkin's lymphoma: experience of a single developing country's oncology centre. PLoS One.

[19] Javanmardi F, Saki-Malehi A, Ahmadzadeh A, Rahim F (2018). Assessing prognostic factors in Hodgkin's lymphoma: multistate illness-death model. Int J Hematol Oncol Stem Cell Res.

[20] Moscona-Nissan A, Mancilla-Osuna MF, Bardán-Duarte A, Rendón-Macías ME (2023). Classical Hodgkin lymphoma histologic subtypes distribution among geographical regions and correlation with Human Development Index. Health Sci Rev.

[21] Iraj AK (2004). Hodgkin's disease: assessment of treatment and survival rates in Iran. Asian Pac J Cancer Prev.

[22] Bhurani D, Nair R, Rajappa S (2021). Real-world outcomes of Hodgkin lymphoma: a multi-centric registry from India. Front Oncol.

[23] Cionini L, Villari N, Ponticelli P, Biti GP, Mungai V (1982). Mediastinal involvement in Hodgkin's disease: prognostic factors and distribution of intrathoracic adenopathies. Eur J Radiol.

[24] Ansell SM (2024). Hodgkin lymphoma: 2025 update on diagnosis, risk-stratification, and management. Am J Hematol.

[25] LaCasce AS (2019). Treating Hodgkin lymphoma in the new millennium: relapsed and refractory disease. Hematol Oncol.

[26] Eichenauer DA, Engert A, André M, Federico M, Illidge T, Hutchings M, Ladetto M (2014). Hodgkin's lymphoma: ESMO clinical practice guidelines for diagnosis, treatment and follow-up. Ann Oncol.

[27] Biasoli I, Castro N, Delamain M (2018). Treatment outcomes for Hodgkin lymphoma: first report from the Brazilian Prospective Registry. Hematol Oncol.

[28] Shafi RG, Al-Mansour MM, Kanfar SS, Al Hashmi H, Alsaeed A, Al-Foheidi M, Ibrahim EM (2017). Hodgkin lymphoma outcome: a retrospective study from 3 tertiary centers in Saudi Arabia. Oncol Res Treat.

[29] Paviglianiti A, Tozatto Maio K, Rocha V (2018). Outcomes of advanced Hodgkin lymphoma after umbilical cord blood transplantation: a Eurocord and EBMT Lymphoma and Cellular Therapy & Immunobiology Working Party Study. Biol Blood Marrow Transplant.

[30] Jacobson CA, Abramson JS (2012). HIV-associated Hodgkin's lymphoma: prognosis and therapy in the era of cART. Adv Hematol.

